# Supplementary material for: Predicting the potential suitable habitats of Rosa roxburghii and its key pest Grapholita molesta in China using the MaxEnt Model
Source: PLoS One. 2026 Apr 24;21(4):e0347592. doi: 10.1371/journal.pone.0347592 (PMC13108782; doi:10.1371/journal.pone.0347592)
Supplement: Attachment — Submitted filename: Response to Reviewers.pdf [file pone.0347592.s006.pdf]

We sincerely thank the Editor and the anonymous reviewers for their constructive and insightful comments, which have greatly helped us to improve the quality and clarity of the manuscript. All comments have been carefully considered and addressed point by point below.

Editor

Comment 1:

Please ensure that your manuscript meets PLOS ONE's style requirements, including file naming. Templates are available at the provided links.

Response to Comment 1:

We appreciate this guidance. The revised manuscript has been formatted according to PLOS ONE style templates. File naming, headings, figure captions, tables, and references have been adjusted to fully comply with the journal’s requirements.

Comment 2:

Provide additional information regarding the permits obtained for the work, including the full name of the authority, or state why no permits were required.

Response to Comment 2:

We thank the editor for this point. All distribution data used in this study were obtained from public databases and published literature. No field sampling was conducted, and no site-specific permits were required. This clarification has been added to the Methods section.

Comment 3:

Ensure that any author-generated code supporting the findings is shared according to PLOS ONE guidelines.

Response to Comment 3:

We acknowledge this requirement. All custom R code used for species distribution modeling and data processing will be deposited in a public repository immediately after the manuscript is accepted, to ensure full reproducibility and reuse. A link to the repository will be provided in the published article.

Comment 4:

Specify the role of funders in the study. If they had no role, state: "The funders had no role in study design, data collection and analysis, decision to publish, or preparation of the manuscript."

Response to Comment 4:

The funders had no role in study design, data collection and analysis, decision to publish, or preparation of the manuscript. This statement has been included in the cover letter and Funding section.

Comment 5:

Ensure that the corresponding author’s ORCID iD is included and validated in Editorial Manager.

Response to Comment 5:
The corresponding author’s ORCID iD has been provided and validated in Editorial Manager.

Comment 6:
Figures 1, 7–16 contain map/satellite images that may be copyrighted. Obtain CC BY 4.0 permission or remove/replace figures.

Response to Comment 6:
We appreciate the editor’s attention to the copyright compliance of the map figures.
All maps used in Figures 1 and 7–16 were generated based on official Chinese standard maps obtained from the Standard Map Service System of the Ministry of Natural Resources of the People’s Republic of China (http://bzdt.ch.mnr.gov.cn/). This platform is publicly accessible and provides standardized maps free of charge. According to the official usage guidelines issued by the Ministry of Natural Resources, these standard maps are compiled following approved national and international boundary delineation standards and may be used for academic publications and other public purposes. Direct use of these maps is permitted provided that the corresponding Map Review Number is clearly indicated. In the revised manuscript, we have explicitly added the Map Review Number in the relevant figures and figure captions.
We note that multiple articles previously published in PLOS ONE have adopted the same practice of using official Chinese standard maps with clearly indicated Map Review Numbers, without reported incompatibility with the CC BY 4.0 license. Examples include:
1. “Spatial-temporal evolution and influencing factors of sudden environmental accidents in China from 2008 to 2022” – map caption clearly indicates Map Review Number GS(2020)4619.
2. “Impacts of key environmental variables on suitable cultivation and flavonoid accumulation in *Pueraria montana* var. lobata under climate change in China” – maps sourced from the official system with Map Review Number GS(2019)1822.
Based on this, we respectfully request confirmation on whether the figures derived from official Chinese standard maps, with Map Review Numbers clearly indicated, can be retained in the manuscript. If these maps are still considered incompatible with CC BY 4.0, we will replace them with maps based entirely on public-domain datasets (e.g., Natural Earth), as recommended.

Comment 7:
Please update your submission to use the PLOS LaTeX template.

Response to Comment 7:
The manuscript has been revised to comply with the journal’s formatting requirements. The revised version is submitted in Word format in accordance with PLOS ONE submission guidelines.

Reviewer #1

Comment 1:

A primary concern is the insufficient optimization of the MaxEnt model parameters. The use of default parameters may lead to excessive model complexity and overfitting. It is strongly recommended to employ R packages like ENMeval to optimize key parameters such as the regularization multiplier and feature classes. The goal should be to select the most parsimonious model, for instance, the one with the lowest delta AICc, and this optimization process should be clearly described in the manuscript. On the topic of environmental variable selection, the current approach is oversimplified. Relying solely on Pearson correlation for variable screening may overlook ecological significance and fails to effectively address multicollinearity. It is suggested to supplement the correlation analysis with techniques like Principal Component Analysis or Variance Inflation Factor analysis. Furthermore, consulting existing literature will help ensure the selected variables have a clear ecological explanation for the species' distributions.

Response to Comment 1:

We appreciate the reviewer's detailed and constructive comments on the model construction methodology. We fully agree that parameter optimization and environmental variable selection in MaxEnt modeling are critical for reducing overfitting and improving predictive reliability, and we have revised the manuscript accordingly to clarify these points.

(1) Optimization of MaxEnt model parameters

In response to the reviewer's suggestions regarding parameter optimization, we systematically tuned the key parameters of the MaxEnt models using the ENMeval package. Specifically, MaxEnt modeling was implemented in the R environment based on the maxnet algorithm, and a comprehensive set of candidate models was evaluated by testing different feature class combinations (L, LQ, and LQH) and regularization multiplier values (1 – 4, with an interval of 0.5). During model evaluation, spatial block cross-validation was applied to reduce the potential influence of spatial autocorrelation on model performance. The small-sample corrected Akaike Information Criterion (AICc) was used as the model selection criterion, and the model with ΔAICc = 0 was selected as the optimal model. As a result, the optimal models for both species were characterized by the LQH feature class combination with a regularization multiplier of 1. These results are now clearly described in the revised manuscript. In addition, the predictive performance of the optimal models was further evaluated using the continuous Boyce index (Fig 3). The Boyce indices for both species were significantly positive, indicating a strong agreement between predicted habitat suitability and observed species occurrences. All of these methodological details and results have been added to the Methods and Results sections of the revised manuscript.

(2) Environmental variable selection

We agree that relying solely on Pearson correlation coefficients may be insufficient to fully address multicollinearity among environmental variables. Therefore, in the revised manuscript, we explicitly clarify that a two-step variable selection strategy was adopted, integrating correlation analysis, multicollinearity diagnostics, and ecological interpretability. First, Pearson correlation coefficients were calculated among the 19 bioclimatic variables, and variable pairs with $|r| > 0.7$

were considered highly correlated. Based on this screening, variables with well-established physiological or ecological relevance in previous studies—such as the minimum temperature of the coldest month (bio6)—were preferentially retained, while other highly correlated variables were removed. Second, variance inflation factor (VIF) analysis was applied to the remaining variables to further diagnose multicollinearity. Only variables with VIF values < 5 were retained for model construction. Compared with the previous screening threshold of $|r| > 0.9$, this revised procedure imposes a more stringent control on multicollinearity while ensuring the retention of key ecologically meaningful predictors. This revised variable selection workflow is now described more clearly in the manuscript.

(3) Ecological interpretation and literature support for environmental variables

Regarding the ecological justification of variable selection, we have further strengthened the biological interpretation of key variables (particularly bio6, bio2, and precipitation-related variables) in the Discussion section. These interpretations are supported by relevant published studies and explain the intrinsic relationships between these variables, the growth characteristics of *R. roxburghii*, and the overwintering biology of *G. molesta*. The corresponding explanations and references have been added and refined in the revised manuscript. In summary, we believe that the revised model parameter optimization procedure and environmental variable selection approach are methodologically robust and ecologically well grounded. We also sincerely thank the reviewer for their valuable suggestions, which have helped to further improve the methodological transparency and scientific rigor of the manuscript.

Comment 2:

For model evaluation and validation, the manuscript currently overrelies on a single evaluation metric, the AUC value. While AUC is useful for measuring discriminatory ability, it is not sensitive to the calibration and reliability of predictions. To provide a more robust assessment, it is advisable to include additional metrics like the continuous Boyce index, which measures the consistency between prediction probabilities and observed distributions. Additionally, conducting spatial cross-validation would better test the model's transferability.

Response to Comment 2:

We thank the reviewer for the constructive suggestions regarding model evaluation. We agree that relying solely on the AUC metric is insufficient to comprehensively assess the reliability and calibration performance of model predictions. In the revised manuscript, we therefore incorporated the continuous Boyce index as a complementary evaluation metric to assess the consistency between predicted suitability values and observed species occurrences. The Boyce index is particularly appropriate for presence – background modeling approaches, such as MaxEnt, and provides a more informative assessment of model reliability. In addition, during model calibration and validation, we applied a spatial block cross-validation approach to reduce the potential influence of spatial autocorrelation on model evaluation and to more effectively assess the spatial transferability of the models. The corresponding methods and results have been added and described in detail in the revised Methods and Results sections.

Comment 3:

In the results analysis and discussion section, there is an insufficient interpretation of the key environmental factor bio6, which is the Min Temperature of Coldest Month. The paper identifies it as a core factor but does not deeply explain its specific biological impact. The discussion should be enhanced by integrating the biological characteristics of both species, such as the low-temperature vernalization requirement for *R. roxburghii* and the overwintering survival rate of *G. molesta*, to elucidate the critical role of bio6 and add significant depth to the analysis.

Response to Comment 3:
We thank the reviewer for pointing out the insufficient ecological interpretation of the key environmental variable bio6 (minimum temperature of the coldest month). We agree that this aspect of the discussion required further elaboration. In the revised manuscript, we have substantially strengthened the discussion of the biological significance of bio6, integrating the ecological and physiological characteristics of both study species. Specifically, for *R. roxburghii*, we expanded the discussion on its sensitivity to winter low temperatures as a perennial woody fruit tree, emphasizing the constraining effects of low temperature on flower bud differentiation and its geographic distribution. For *G. molesta*, we further clarified the importance of extreme low-temperature conditions for overwintering survival and population persistence, considering its biological trait of overwintering in larval or pupal stages. By incorporating these ecological and physiological mechanisms, the limiting role of bio6 as a core environmental factor has been more thoroughly and explicitly explained in the revised Discussion section.

Comment 4:
Concerning the presentation of results, the use of a single future climate change scenario, SSP5-8.5, limits the generalizability of the conclusions. If feasible, it is recommended to incorporate simulations for one to two different emission pathways, such as SSP1-2.6 or SSP2-4.5. Analyzing the uncertainty of distribution changes across different scenarios would make the study more comprehensive and its findings more robust.

Response to Comment 4:
The reviewer raised an important point regarding the selection of future climate scenarios. Relying on a single high-emission scenario may limit the generality of the conclusions. In the revised manuscript, the future projections were therefore expanded by adding the SSP2-4.5 intermediate-emission scenario in addition to the original SSP5-8.5 scenario. A comparative analysis of the projected changes in potential suitable habitats for *R. roxburghii* and *G. molesta* was conducted under both scenarios. By contrasting results across multiple emission pathways, the uncertainty associated with species distribution responses to different climate trajectories was more comprehensively evaluated, thereby improving the robustness and applicability of the study conclusions. The relevant results and interpretations have been incorporated into the revised Results and Discussion sections.

Comment 5:
For language and formatting, some minor errors were noted, such as repeated periods in the abstract and main text. A thorough round of language polishing is recommended before submission to ensure professionalism and fluency. Simultaneously, please strictly check and adhere to the PLoS ONE

journal's specific formatting requirements for submissions.

Response to Comment 5:

We appreciate the reviewer’s comments regarding language and formatting. In response, the entire manuscript was carefully revised for language clarity and consistency, and minor issues such as repeated punctuation in the abstract and main text were corrected. In addition, the manuscript was thoroughly checked and adjusted in strict accordance with the PLOS ONE submission guidelines, including the structure, figures, tables, references, and overall formatting, to ensure professionalism and compliance with journal standards.

Reviewer #2

Comment 1:

Insufficient clarity on the literature data.

The manuscript does not specify the temporal scope of the literature survey (i.e., which years were covered). In addition, the number of valid/eligible publications included after screening should be explicitly reported. This information is essential for evaluating data completeness and potential bias.

Response to Comment 1:

We appreciate the reviewer's valuable comments. In this study, the systematic literature survey was conducted primarily for *G. molesta*, as the occurrence records of *R. roxburghii* available in public databases were already sufficiently abundant to meet the analytical requirements of this study, and therefore an additional systematic literature search for *R. roxburghii* was not performed. For *G. molesta*, literature searches were carried out in both the Web of Science and China National Knowledge Infrastructure (CNKI) databases. The temporal scope of the search was restricted to publications from 1970 to the present, consistent with the baseline period (1970 – 2000) of the climate data used in this study. The initial search yielded a total of 2,772 publications (786 from Web of Science and 1,986 from CNKI). These records were then screened on a study-by-study basis according to clearly defined inclusion and exclusion criteria, resulting in 36 publications that contained explicit records of the natural distribution of *G. molesta* and were retained for subsequent analyses. All of the above information has been clearly described in the revised Methods section and summarized in the Supporting Information (S1 Table).

Comment 2:

Paragraph duplication in "Species Distribution Data."

In the Materials and Methods section (Species Distribution Data), the second and third paragraphs are nearly identical. Both repeat the same six filtering criteria for occurrence records with only minor wording differences. This appears to be a clear editing or copy–paste error. Please remove one of the duplicated paragraphs and carefully check the manuscript to ensure no similar repetitions remain elsewhere.

Response to Comment 2:

We are grateful to the reviewer for identifying the paragraph duplication in the Species Distribution Data section. Upon careful review, the second and third paragraphs were indeed found to be highly repetitive due to an editorial oversight. In the revised manuscript, the redundant paragraph has been removed, and a single, consolidated description of the distribution data screening criteria has been retained. In addition, the entire manuscript was systematically checked to ensure that no similar duplication or formatting errors remain. The reviewer's attention to this detail is sincerely acknowledged.

Comment 3:

Unclear map-related statement.

Please clarify whether the manuscript should report a map survey number or a map approval number for China map.

Response to Comment 3:

The reviewer raised a concern regarding the source and compliance of the map base layers used in this study. The China base map adopted in the manuscript was obtained from the National Platform for Common GeoSpatial Information Service of China, which provides officially released standard maps compiled in accordance with national and international boundary delineation standards. These standard maps are permitted for public uses such as academic publications, book illustrations, and the presentation of scientific results, and are freely accessible to the public. According to the platform's usage regulations, the Map Review Number must be indicated when standard maps are directly used. Accordingly, the corresponding Map Review Numbers have now been clearly labeled in the revised figures (e.g., Fig 1), and this information has been added to the figure captions to improve transparency and ensure compliance with map usage requirements.

Comment 4:

Overly generic protection strategies.

The section on Protection Strategies remains too broad and descriptive. The authors should provide more concrete, feasible, and actionable recommendations (e.g., targeted monitoring in projected overlap hotspots, region-specific management timelines, or practical cultivation/pest-control adjustments under future climate scenarios). Strengthening this part would improve the applied value of the study.

Response to Comment 4:

Thank you for the reviewer's valuable comments. We agree that the "Protection Strategies" section in the original manuscript was relatively general in its description, and that its practical specificity required further improvement. Following the reviewer's suggestions, we have revised and refined this section to make the management recommendations more concrete, feasible, and directly linked to the model results. After revision, the protection strategies are explicitly based on the overlap analysis of suitable habitats between *R. roxburghii* and *G. molesta*, and the following specific measures are proposed: (1) high-suitability overlap hotspots identified under current climate conditions (e.g., northeastern Sichuan and surrounding areas) are designated as priority monitoring regions, where intensified field surveys and overwintering population monitoring are recommended and incorporation into long-term early-warning systems is suggested; (2) under future climate scenarios, in light of the northward shift in the potential distribution of *G. molesta*, newly expanded or potential cultivation areas of *R. roxburghii* should be proactively included in pest risk assessment and monitoring frameworks to prevent emerging infestations driven by newly suitable climatic conditions; and (3) at the cultivation and pest management level, management strategies should be adjusted according to regional warming trends by optimizing cultivar selection (e.g., heat-tolerant or low-chilling-requirement varieties), improving planting layout, and integrating biological control and ecological regulation measures to enhance the stability and resilience of the *R. roxburghii* production system. Through these revisions, the protection and management recommendations have shifted from a broad conceptual framework to region-specific and scenario-based strategies grounded in spatial prediction results, thereby substantially enhancing the applied value and practical relevance of the study.

Reviewer #3

Comment 1:
Terminology: The term “predation” is used to describe the feeding behavior of *G. molesta* on *R. roxburghii*. In ecological terminology, predation refers specifically to animals killing and consuming other animals. In this case, the correct term is “herbivory.” The terminology should be corrected throughout the manuscript to avoid conceptual confusion.

Response to Comment 1:
The reviewer identified an important issue regarding the use of ecological terminology. In ecology, the term predation specifically refers to animals feeding on other animals, whereas the interaction between *G. molesta* and *R. roxburghii* should be more accurately described as herbivory. Accordingly, in the revised manuscript, all instances referring to the relationship between *G. molesta* and *R. roxburghii* have been consistently corrected from “predation” to “herbivory” to avoid conceptual ambiguity and to ensure accurate use of ecological terminology.

Comment 2:
Background on *G. molesta*: The introduction lacks sufficient background on *G. molesta*, a pest that has been extensively studied, particularly in China. The authors are encouraged to expand the introduction to include: Known distribution patterns of *G. molesta*； Typical dispersal pathways (including the role of trade and human-mediated movement)； Differences between infested and non-infested regions； Its host range, as *G. molesta* attacks multiple fruit crops and is not restricted to *R. roxburghii*. Providing this context is important for interpreting the ecological and management implications of the distribution overlap results.

Response to Comment 2:
The reviewer’s comment highlighted the need for a more comprehensive background on *G. molesta*. As an economically important fruit pest that has been extensively studied in China and worldwide, additional contextual information was warranted in the Introduction. In the revised manuscript, the Introduction has been substantially expanded to include:
(1) the known distribution patterns of *G. molesta* in China and globally; (2) its major dispersal pathways, with particular emphasis on the role of human-mediated activities such as fruit trade and the transport of nursery stock; and (3) its broad host range, highlighting that *G. molesta* is not restricted to *R. roxburghii* but is capable of feeding on multiple fruit tree crops. These additions provide clearer ecological context for understanding the potential overlap in suitable habitats between *R. roxburghii* and *G. molesta*, as well as the associated management implications.

Comment 3:
The selection criteria for environmental variables for both the pest and the host plant appear nearly identical and could be presented together to reduce redundancy.

Response to Comment 3:
We thank the reviewer for pointing out the redundancy in the section on environmental variable

selection. We agree that, given the high similarity in the variable selection procedures for *R. roxburghii* and *G. molesta*, presenting them in a unified manner improves conciseness and clarity. Accordingly, in the revised manuscript, we have restructured this section by combining the environmental variable selection criteria and analytical workflow for both species, while highlighting species-specific differences in responses to key variables where necessary. This revision reduces repetition and enhances the overall readability of the manuscript.

Comment 4:

The manuscript states that variables with correlation coefficients greater than 0.9 were excluded. This threshold is relatively permissive, and highly correlated variables may still enter the model. The authors should: 1) provide references supporting this threshold, or 2) further justify their choice of variables based on ecological knowledge and previous modeling studies. 3) relevant literature on variable selection in distribution modeling for *R. roxburghii* and *G. molesta* should be cited.

Response to Comment 4:

The reviewer raised an important point regarding the choice of correlation thresholds and the robustness of the environmental variable selection procedure. We agree that relying solely on an initial cutoff of $|r|>0.9$ is relatively permissive and may allow highly correlated variables to remain in the model, thereby increasing the risk of multicollinearity. In response, we have clarified and strengthened the variable selection workflow in the revised manuscript as follows:

Correlation screening:

Pearson correlation coefficients were calculated among the 19 bioclimatic variables, and pairs with $|r|>0.7$ were considered highly correlated. This threshold is commonly adopted in species distribution modeling to balance multicollinearity control and information retention, and it provides a more stringent identification of potential collinearity than the previously stated $|r|>0.9$.

Integration of ecological relevance:

After the initial screening, variables were further evaluated based on species-specific ecological and physiological relevance. Priority was given to variables with clear biological meaning or known threshold effects (e.g., minimum temperature of the coldest month, bio6), while other variables highly correlated with these key predictors were removed to maintain model interpretability and biological plausibility.

Collinearity diagnosis:

Variance inflation factor (VIF) analysis was subsequently applied to the retained variables, and only predictors with $VIF < 5$ were included in the final model to further reduce multicollinearity. Through these revisions, the environmental variable selection procedure is now more rigorously controlled statistically while being firmly grounded in ecological considerations, thereby improving the robustness and interpretability of the model.

Comment 5:

The phrase “The survey map number” appears to be incorrect and should be revised to “Map Review Number.”

Response to Comment 5:

The reviewer pointed out an issue regarding the use of map-related terminology. Following this suggestion, the term “The survey map number” has been consistently revised to “Map Review Number” throughout the manuscript, and the figure captions have been standardized accordingly.

Comment 6:

Under current climate conditions, the overlap between high suitability areas (HSA) of *R. roxburghii* and *G. molesta* is very limited. This suggests that *G. molesta* may not pose a widespread threat across the entire suitable range of *R. roxburghii*.

Response to Comment 6:

We appreciate this insightful comment on the limited overlap of high-suitability areas under current climatic conditions. This result indicates that *G. molesta* does not pose a widespread threat across the entire potential suitable range of *R. roxburghii* at present. In the revised Discussion, the ecological implications of this finding are further emphasized, highlighting that the potential risk of *G. molesta* is currently concentrated in localized regions rather than being pervasive at the national scale.

Comment 7:

Figures 12 and 13 appear redundant; Figure 13 alone would be sufficient.

Response to Comment 7:

We appreciate the suggestion regarding the presentation of figures. While concerns were raised about potential redundancy, it is important to clarify that Figures 12 and 13 convey different aspects of risk. Figure 12 illustrates the spatial overlap of *R. roxburghii* and *G. molesta* at the high suitability area (HSA) level, highlighting core regions with the highest potential risk under current and future scenarios that require priority attention and management. Figure 13, on the other hand, extends this by incorporating the moderate suitability area (MSA), reflecting regions that may experience intermediate-level risk under climate change. Each figure corresponds to distinct risk levels and management contexts, providing complementary insights. In the revised manuscript, rather than removing either figure, we have reorganized and integrated the presentation of distribution results across different time periods and climate scenarios to reduce visual redundancy and improve overall clarity and aesthetic presentation, while still preserving the distinction and comparability of areas across suitability levels. Figure captions and relevant text have also been updated accordingly to prevent potential confusion. These revisions maintain the integrity of the study while addressing concerns regarding figure clarity and readability.

Comment 8:

8. The authors may consider merging distribution maps into composite panels (e.g., current vs. future scenarios) to reduce repetition and improve visual clarity.

Response to Comment 8:

We appreciate the suggestion regarding the integration of figures. In response, selected distribution maps have been reorganized into composite panels, combining current and future scenario results to reduce redundancy and enhance visual clarity.

Comment 9:
9. Discussion Clarity: Some statements in the discussion are potentially confusing. For example, the manuscript states that the "HSA of *R. roxburghii* is similar in size to that of *G. molesta*," while earlier results indicate large differences in total suitable area. If this comparison refers specifically to high suitability areas, this should be stated explicitly to avoid misunderstanding.

Response to Comment 9:
We appreciate the comment regarding potential ambiguity in the Discussion section. In the revised manuscript, it is now clearly stated that comparisons of the suitable area sizes between *R. roxburghii* and *G. molesta* specifically refer to high suitability areas (HSA) rather than the total suitable area. Corresponding sentences have been reworded to prevent any misunderstanding.

Comment 10:
10. The discussion of limited HSA overlap could be strengthened by emphasizing its ecological and management implications. Given that the overlapping HSA represents only 0.33% of China's land area, these regions may represent priority hotspots for monitoring and targeted pest management.

Response to Comment 10:
We greatly appreciate the insightful suggestion regarding the management implications of the overlap in high suitability areas (HSA). While the overlapping HSA accounts for only 0.3% of China's land area following the methodological refinements, its ecological and management significance remains notable. In the revised manuscript, this finding is further emphasized in the Discussion, highlighting that these overlapping regions can be considered priority areas for future monitoring and targeted management of *G. molesta*. Recommendations for implementing dynamic surveillance and focused control measures in these areas are provided, thereby enhancing the practical relevance of the study.
