# Supplementary material for: Predicting the potential suitable habitats of Rosa roxburghii and its key pest Grapholita molesta in China using the MaxEnt Model
Source: PLoS One. 2026 Apr 24;21(4):e0347592. doi: 10.1371/journal.pone.0347592 (PMC13108782; doi:10.1371/journal.pone.0347592)
Supplement: Attachment — Submitted filename: Response to Reviewers.docx [file pone.0347592.s007.docx]

We are grateful to the Editor and Reviewer #3 for their valuable and thoughtful feedback, which has significantly enhanced the clarity and overall quality of our manuscript. We have thoroughly considered each comment and provide detailed, point-by-point responses below.

**Reviewer #3**

Comment 1:

Line 24: Expressing the distribution as “0.3% of the country” is not very informative. If the intention is to emphasize the limited distribution of this species, it would be clearer to report the absolute area. Given that this species is mainly restricted to regions of southwest China, providing exact area values would improve clarity.

Response to Comment 1:

Thank you for this helpful suggestion. We agree that expressing the distribution solely as a percentage does not clearly convey the limited extent of the species’ range. Following your recommendation, we have revised the manuscript to include the absolute area of overlap. Specifically, we now report that the highly suitable overlapping area between the two species is approximately 28,800 km², while retaining the percentage (~0.3% of China’s land area) for additional context. This revision provides a clearer representation of the spatial extent and emphasizes the restricted nature of the potential high-risk areas (**Lines 23–29 in the Manuscript, the corresponding revisions are highlighted in yellow in the Revised Manuscript with Track**).

Comment 2:

Line 43-45: The meaning is somewhat unclear. The term “overlapping ecological niches” is not appropriate here and could be replaced with “co-occurrence”. The statement also conflates climatic effects with niche overlap, as pest–crop interactions already imply spatial co-occurrence; climate primarily modulates their distribution and interaction intensity rather than determining overlap per se.

Response to Comment 2:

Thank you for this insightful comment. We agree that the term “overlapping ecological niches” was not appropriate in this context and that our original wording conflated niche overlap with climatic effects. Following your suggestion, we have revised the text to use “co-occurrence” and clarified the role of climate as a factor that modulates the likelihood of co-occurrence and the intensity of pest–crop interactions, rather than determining overlap itself. (Lines 46–48).

Comment 3:

Line 52: The use of “globally” is inappropriate here; “worldwide” or “across the world” would be more appropriate

Response to Comment 3:

Thank you for this suggestion. We have revised “globally” to “worldwide” in the manuscript (Line 56).

Comment 4:

Line 134-139: Please clarify the source of the data. If a website was used, the full URL and access date should be provided.

Response to Comment 4:

Thank you for this helpful suggestion. We have clarified the data source by providing the full URL along with the access date in the revised manuscript (Line 139): (https://www.tianditu.gov.cn/

, accessed in July 2025).

Comment 5:

Line 204-207: "areas above the threshold were further divided into Low Suitability Areas (LSA), Moderate Suitability Areas (MSA), and High Suitability Areas (HSA). " Please clarify how this classification was performed. Were these categories defined using equal intervals or another thresholding method?

Response to Comment 5:

Thank you for this helpful comment. We have clarified that the classification was performed using an equal-interval approach. Specifically, values above the threshold were divided into three categories (LSA, MSA, and HSA) by equally partitioning the range between the threshold and the maximum suitability value (1). This clarification has been added to the revised manuscript (Lines 208–211).

Comment 6:

Line 214-247: The description of the current potential distribution is overly detailed and somewhat difficult to follow. Listing numerous province names does not necessarily improve clarity, especially for an international readership unfamiliar with China’s administrative regions. I suggest simplifying the presentation by focusing on key patterns rather than exhaustive geographic enumeration. For example, the authors could (1) report the total suitable area, (2) summarize the spatial pattern using broader geographic descriptors (e.g., southwestern, central, or eastern China), and (3) highlight where high-suitability areas are concentrated along with their corresponding area values. This more structured and synthetic description would make the results clearer and more informative.

Response to Comment 6:

We agree that the original description was overly detailed and not sufficiently reader-friendly, particularly for an international audience. Following your recommendation, we have substantially revised this section by removing excessive province-level enumeration and reorganizing the presentation. In the revised version, we now (1) report the total suitable area, (2) summarize the overall spatial patterns using broader geographic descriptors (e.g., southwestern, central, and eastern China), and (3) highlight the distribution and extent of high-suitability areas along with their corresponding area values. These changes improve the clarity, conciseness, and overall readability of the results section.

Comment 7:

Line 296-310: The use of “percentage of China’s land area” is not very informative for assessing ecological or agricultural significance. It would be more meaningful to report absolute area values and/or the proportion of overlap relative to the total suitable area of the host plant. In addition, the comparison based on high-suitability overlap (HSA) is potentially misleading. Pest damage can occur wherever the pest and host co-occur, regardless of suitability class, and areas with moderate suitability may still support populations capable of causing substantial yield loss. I suggest simplifying the analysis by focusing first on the overall spatial overlap between pest and host suitable areas, and then evaluating how this overlap is distributed across the host’s suitability gradient. This would provide a clearer and more ecologically meaningful assessment of potential risk.

Response to Comment 7:

Thank you for this insightful and constructive comment. We agree that using percentages alone is not sufficient to reflect the ecological and agricultural significance of the results. In response, we have revised the manuscript to report absolute area values in place of, and in addition to, percentages.

Following your suggestion, we have also refined the analysis and interpretation by not limiting our focus to high-suitability overlap (HSA). In the revised manuscript, we first describe the general pattern of overlap and then provide a concise interpretation of its potential ecological implications for *R. roxburghii*. This approach is now reflected in the revised Results (Lines 293–317).

In addition, we have strengthened the Discussion to clarify that overlap in suitable areas represents the potential for co-occurrence rather than direct damage, and therefore should not be interpreted as a direct measure of pest impact. We also explicitly note that most of the overlap occurs within low-suitability areas (LSA), indicating that the host plant still has a large spatial extent where it may be exposed to pest pressure, although the probability of occurrence is relatively low. Furthermore, although the overlap in high-suitability areas (HSA) remains limited, these regions may represent relatively higher-risk zones. (Lines 374–392).

Comment 8:

Line367-371, 411: The interpretation appears to assume that spatial overlap directly reflects pest impact, which is not necessarily valid. As noted above, overlap does not equal impact strength, and this distinction should be more clearly addressed.

Response to Comment 8:

Thank you for this helpful comment. We agree that spatial overlap should not be interpreted as direct pest impact. In the revised manuscript, we have explicitly clarified this point in the Discussion (Lines 374–377), where we state that the term “potential threat” refers to the possibility of spatial co-occurrence between the pest and the host, rather than actual pest damage. This clarification helps to avoid overinterpretation of the overlap results and ensures a more accurate ecological interpretation of the findings.

Comment 9:

The conclusion is overly long and reads more like a condensed repetition of the Results and Discussion sections. It would benefit from a more concise and focused synthesis of the key findings. I suggest restructuring this section to clearly highlight (1) the main objective, (2) two to three key findings, and (3) the primary implication of the study.

Response to Comment 9:

Thank you for this helpful suggestion. We agree that the original conclusion was overly long and largely repetitive of the Results and Discussion sections. Following your recommendation, we have substantially revised and restructured the conclusion to make it more concise and focused.

In the revised version, we first clearly state the main objective of the study, then summarize three key findings in a more structured manner, and finally highlight the primary implication regarding the spatial pattern of potential pest risk under current and future climate scenarios. These revisions improve the clarity and synthesis of the conclusion while avoiding unnecessary repetition of detailed results.
